# PHYSIO-FEEDBACK EXERCISE PROGRAM (PEER) ON ACCELEROMETER-BASED DATA: A SMOOTHING SPLINE ANOVA MODEL

**DOI:** 10.1093/geroni/igae098.4046

**Published:** 2024-12-31

**Authors:** Chang Liu, Yi Liu, Ladda Thiamwong, Tho Nguyen, Wei Zhang, Rui Xie

**Affiliations:** University of Central Florida, Orlando, Florida, United States; University of Central Florida, Orlando, Florida, United States; University of Central Florida, Orlando, Florida, United States; University of Central Florida, Orlando, Florida, United States; University of Central Florida, Orlando, Florida, United States; University of Central Florida, Orlando, Florida, United States

## Abstract

Exercise is crucial for reducing falls in older adults. Physio-fEedback Exercise pRogram (PEER) consists of physio-feedback, cognitive reframing, and peer-led exercises that can reduce fall risk. However, there is a lack of data on the outcome of PEER on physical activity (PA) that may provide valuable insights for immediate and long-term improvements. We examined the effectiveness of PEER intervention on the daily PA level from 64 low-income older adults (mean age = 73.1 ± 6.26 years, female 87.5%), randomized into either PEER or control groups. The PEER group participated in weekly exercise sessions led by a trained peer for 8 weeks, followed by 13 weeks of follow-up, while the control group data was collected over a similar 21-week period without intervention. Physical activity was measured using a commercial accelerometer (Fitbit device). Data were categorized into sedentary, lightly active, moderately active, and very active minutes. We evaluated the peer-led group exercise days on four daily activity levels using a Smoothing Spline ANOVA (SSANOVA) model with partial splines and incorporated parameter covariates into the classic SSANVOA model. The results indicated a significant increase in moderately active and very active minutes in the PEER group during the 8-week intervention period, highlighting the short-term effectiveness of the program. We also observed the attenuation of the intervention’s effects over the 13-week follow-up across all four activity levels. PEER intervention significantly boosts daily activity levels among older adults in the short term, and sustained efforts may be necessary to maintain these benefits over the long term.

